# Epidemiology of Match Injuries in Portuguese Senior Male Rugby Union XV: A Prospective Cohort Study

**DOI:** 10.3390/medicina62081501

**Published:** 2026-08-05

**Authors:** Carlos Braga, Nuno Neves da Costa, Júlio A. Costa, António Cruz-Ferreira

**Affiliations:** 1 Faculdade de Medicina, Universidade de Coimbra, 3004-531 Coimbra, Portugal; nnevesdacosta@gmail.com (N.N.d.C.); krusferreira@hotmail.com (A.C.-F.); 2 Centro de Estudos de Investigação em Saúde, Universidade de Coimbra, 3004-531 Coimbra, Portugal; 3 Federação Portuguesa de Rugby, 1600-007 Lisboa, Portugal; 4 FPF Academy, Portuguese Football Federation, Cidade do Futebol, Avenida das Seleções, 1495-433 Oeiras, Portugal; julio.costa@fpf.pt; 5 Departamento de Medicina, Instituto do Desporto e Juventude, 1250-190 Lisboa, Portugal

**Keywords:** contact sport, trauma, sports medicine, severity, prevention

## Abstract

*Background and Objectives*: Rugby Union is associated with a high incidence of injuries. Although injury epidemiology has been extensively documented internationally, data from Portuguese rugby remain scarce despite the sport’s recent growth and increasing visibility. This study aimed to investigate the incidence, characteristics, and severity of time-loss match injuries, as well as their impact on match availability, in the top tier of Portuguese Rugby Union XV. *Materials and Methods*: A prospective cohort study was conducted during the 2023/2024 Portuguese rugby season. Five of the ten eligible teams competing in the Portuguese Senior Male Rugby Union XV Championship participated in the study. Team medical staff prospectively recorded all time-loss match injuries using a standardized online reporting form. *Results*: The overall injury incidence was 49.46 injuries per 1000 player-match-hours (95% CI: 39.78–59.68), with a mean injury severity of 12.80 days (SD = 12.17). Lower-limb injuries accounted for 52.2% of all injuries and were associated with the greatest mean time-loss (15.56 days). Bone injuries demonstrated the highest descriptive mean injury severity. However, this finding was based on a small number of cases and should therefore be interpreted with caution. A weak but statistically significant positive correlation was observed between exposure hours and injury severity (r = 0.26, *p* = 0.011). *Conclusions*: The findings indicate that injuries in Portuguese top-tier rugby occur predominantly during contact events and in the second half of matches. Lower-limb injuries were the most frequently reported injuries and accounted for the largest proportion of time-loss injuries.

## 1. Introduction

Rugby distinguished itself not only as a high-contact sport involving physical elements such as tackling and scrummaging, but also through its deeply rooted values of teamwork, camaraderie, and mutual respect among players and officials [1]. These characteristics have contributed to the development of a distinctive culture both on and off the field, fostering a strong sense of community among athletes and supporters alike.

In Portugal, Rugby Union has experienced considerable growth over the past decade. The national team’s performance at the 2023 Rugby World Cup attracted significant attention, and during the 2023/2024 season, the Portuguese top-tier final was broadcast live on national television for the first time in the sport’s history.

At both elite and amateur levels, Rugby Union is associated with a substantial risk of injury [2,3]. Injuries are not only frequent but can also be severe, resulting in prolonged absences from participation and potentially long-term consequences for athletes [3]. A study conducted during the 2022/2023 Portuguese season reported an overall injury incidence of 54.4 injuries per 1000 player-match-hours [4]. Injury incidence appears to be even higher in elite competitions. For example, the England Professional Rugby Injury Surveillance Project reported an overall incidence of 78 injuries per 1000 player-match-hours during the 2022/2023 season [5]. In comparison with many other sports, rugby demonstrates a considerably higher injury incidence [6]. In professional football, for instance, the UEFA Elite Club Injury Study, which monitored men’s professional football between the 2000/2001 and 2018/2019 seasons, reported an overall injury incidence ranging from 20 to 35 injuries per 1000 player-match-hours [7].

Among rugby players, forwards are more likely to sustain collision-related injuries, such as fractures and joint dislocations, due to the high levels of physical contact encountered during match play [4]. In contrast, backs appear more susceptible to muscular and joint injuries, reflecting the frequent acceleration, deceleration, changes in direction, and sidestepping actions required in their positional roles [8]. These injury patterns have become a growing concern for players, coaches, administrators, and medical staff alike [2,5,9]. Given the substantial injury burden associated with the sport and its potential impact on athlete welfare, understanding injury epidemiology is essential for informing effective prevention strategies.

As injury data from Portuguese rugby remain limited, this study aimed to investigate and describe the epidemiology of injuries sustained in the Portuguese top-tier Rugby Union competition during the 2023/2024 season and to compare the findings with those reported in other rugby populations. Improved understanding of injury patterns may contribute to the development of evidence-informed prevention strategies aimed at reducing injury incidence and promoting player health and safety.

## 2. Materials and Methods

Ethical approval was obtained from the Ethics Committee of the local Regional Health Administration, and institutional collaboration was granted by the Portuguese Rugby Federation.

Portugal’s “Top 10” consisted of ten teams during the 2023/2024 season. The competition was divided into two phases: the regular season and the play-offs. During the regular season, each team played against every other team on a home-and-away basis (18 matches per team in total). At the end of the regular season, the top six teams progressed to the play-offs, while the bottom four teams did not play any further matches (with the team finishing in 10th place being relegated to the second tier). Among the top six teams, those finishing in first and second place advanced directly to the semi-finals, where they faced the winners of the quarter-finals (contested by the teams that finished in third to sixth place). The Portuguese champions were crowned in a final match contested by the winners of the two semi-finals.

### 2.1. Participants

All ten teams were invited to participate in the study and were provided with a set of information detailing the study methodology, objectives, procedural requirements, and the agreed terminology necessary for accurate injury documentation [10].

**Figure 1:**
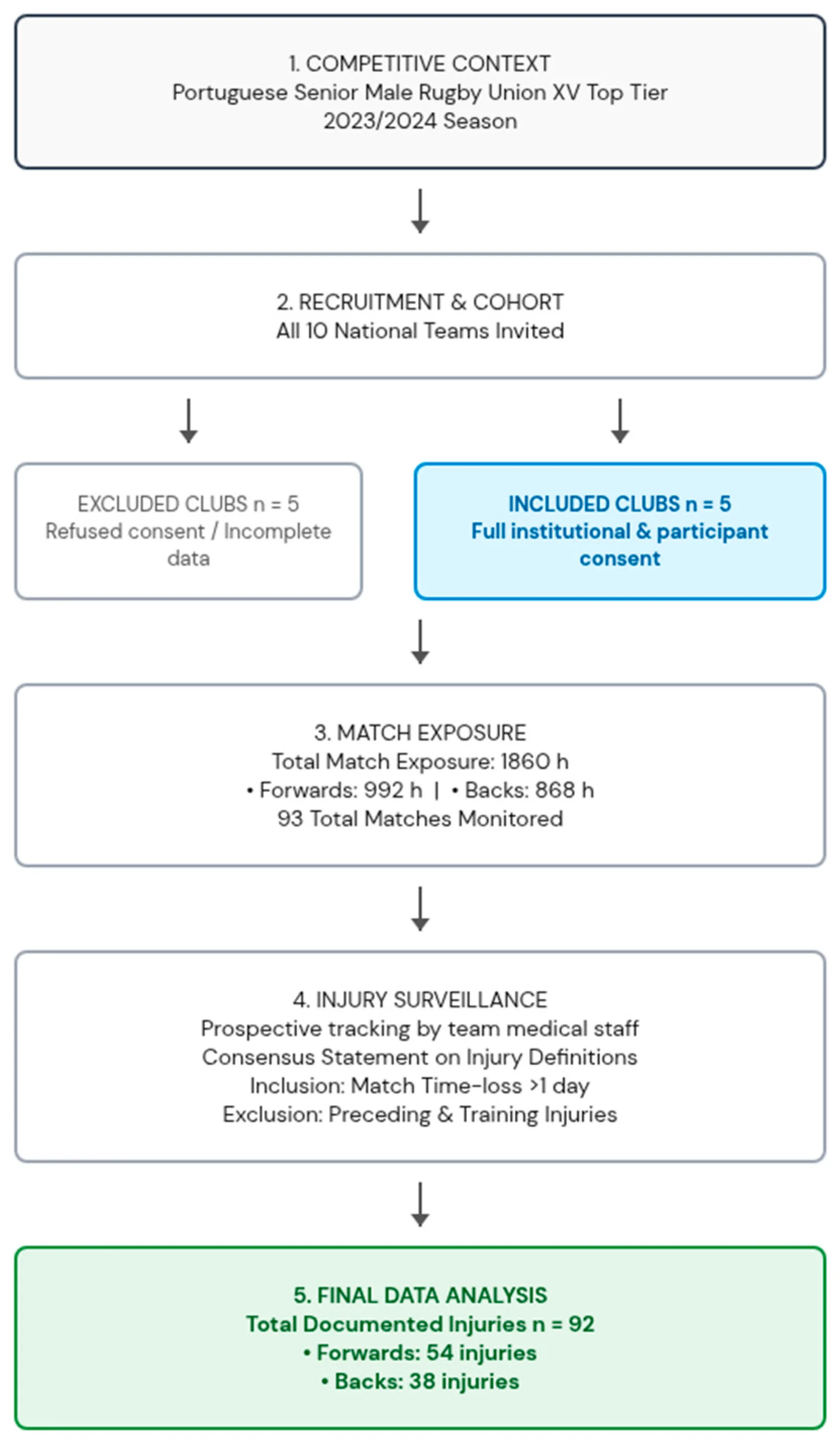
Diagram of Materials and Methods.

Written informed consent was obtained from all participants prior to data collection, in accordance with the requirements of the institutional review board. The participants were assured of confidentiality, and all data were anonymised to protect their identities. No invasive procedures were involved, and participation was voluntary, with the right to withdraw at any time without consequence. A resume of all of this information is presented in Figure 1.

### 2.2. Injury Definitions

The term “injury” was defined according to the international Consensus Statement on Injury Definitions and Data Collection Procedures for Studies of Injuries in Rugby Union as “any physical complaint sustained by a player during a match that prevented the player from taking a full part in training and/or match play for more than one day following the day of injury, regardless of whether matches or training sessions were scheduled” [11]. The term “severity” was defined as the number of days between the occurrence of the injury and the player’s full recovery, excluding both the day on which the injury occurred and the day on which the player returned. A player was considered to have regained full fitness when he was able to participate fully in training activities and was available for match selection.

### 2.3. Exposure

Injury incidence was calculated as the number of injuries per 1000 player-match-hours (injuries/match exposure × 1000; 95% CI). Match exposure was estimated using a team-based approach, assuming full participation of 15 players per team throughout each team-match exposure (15 players × number of team-match exposures × 80 min). Positional exposure was estimated assuming eight forwards and seven backs per team. No adjustments were made for temporary player removals, disciplinary sanctions, injuries occurring during matches, or periods during which teams competed with fewer than 15 players because reliable and standardised player-level time-on-field data were not available across all the participating teams. Although this approach may slightly overestimate actual exposure, it was applied consistently across all the participating teams, allowing valid internal comparisons of injury incidence and burden estimates.

The injury burden was calculated as days lost per 1000 player-match-hours (total days lost/exposure hours × 1000; 95% CI). Confidence intervals were estimated using bootstrapping methods as described by Williams et al. [12].

### 2.4. Study Design

A prospective cohort study was conducted during the 2023/2024 season in Portugal’s top-tier Rugby Union competition. Injury surveillance, data collection procedures, injury classification, and reporting followed the international consensus recommendations for Rugby Union injury surveillance [11,13]. The surveillance procedures adopted in the present study were also consistent with those implemented in previous Rugby Union epidemiological investigations, including Portuguese cohorts, thereby facilitating comparison with the existing literature [4].

### 2.5. Data Collection

Before the beginning of the competitive season, all the participating medical staff received standardized instructions regarding injury definitions, classification criteria, and data collection procedures to ensure consistency across teams. Throughout the study period, injury registration within each club was performed by the same designated healthcare professional whenever possible, thereby reducing intra-team variability in injury reporting and classification.

Each team medical staff member was responsible for registering all the injuries that occurred during matches as well as the specifications requested (player position, date of injury, pitch location, match time, anatomical location, type of injury, relation with contact, body side and return-to-play date). Only injuries sustained during the surveillance period were included in the analysis. Injuries that occurred before the start of the study were not recorded. Players who were unavailable because of pre-existing injuries entered the surveillance cohort only after returning to unrestricted participation. Subsequent injuries occurring during the surveillance period were recorded according to the study protocol. Training-related injuries were not included due to the lack of consistent and reliable recording methods across teams during training sessions. As match data were standardised and systematically reported by medical staff, only match-related injuries were analysed to ensure data accuracy and comparability.

### 2.6. Statistical Analysis

Statistical analyses were performed using appropriate parametric and non-parametric procedures according to data type and distribution. Continuous variables, such as injury severity (days lost), were analyzed using one-way ANOVA. When assumptions of homogeneity of variance were violated, Games-Howell post hoc tests were applied for pairwise comparisons. Categorical variables, including anatomical location, injury type, contact mechanism, and body side, were analyzed using chi-square tests. Fisher’s exact test was used when expected cell counts were less than five. Injury incidence and injury burden were calculated as rates per 1000 player-match-hours, with 95% confidence intervals estimated using bootstrapping methods. The level of statistical significance was set at *p* < 0.05.

## 3. Results

All ten clubs competing in the Portuguese Senior Male Rugby Union XV Championship were invited by email to participate in the study. Five clubs agreed to participate and distributed the study invitation to their registered senior male players. Players who agreed to participate completed the study questionnaire and provided written informed consent before enrolment. Only participants who completed both the questionnaire and informed consent were included in the injury surveillance database, and only injuries sustained by these players were recorded and analysed. Among these participating teams, three did not qualify for the playoffs, one reached the quarterfinals, and one was the league champion.

In contrast, the teams that did not participate were generally higher-ranked in the league and had greater access to medical resources, including more structured medical staffing. This difference should be considered when interpreting the representativeness of the sample, as it may have introduced a selection bias.

The total match exposure among participating teams was 1860 player-match-hours (forwards: 992 h; backs: 868 h), corresponding to 93 team-match exposures. Across these team-match exposures, a total of 92 injuries were recorded (forwards: 54; backs: 38) (Table 1).

The overall injury incidence was 49.46 injuries per 1000 player-match-hours (95% CI: 39.78–59.68). When analysed according to playing position, forwards presented an injury incidence of 54.44 injuries per 1000 player-match-hours (95% CI: 40.32–69.56), while backs showed an incidence of 43.78 injuries per 1000 player-match-hours (95% CI: 29.95–57.60). Forwards sustained most injuries (58.7%), and the overall mean injury severity was 12.80 days (SD = 12.17), although it was slightly higher among backs (13.63 days). The overall injury burden was 633.33 days lost per 1000 player-match-hours (665.32 in forwards; 95% CI: 614.92–714.72, and 596.77 in backs; 95% CI: 546.08–647.47) (Table 1).

More than half of all injuries affected the lower limb (52.2% of the total number of injuries). When analysed by playing position, the lower limb was also the most frequently injured body region (48.1% in forwards and 57.9% in backs), with no statistically significant difference between positions (*p* = 0.329) (Table 2). Regarding injury type, joint/ligament injuries were the most prevalent (32.6%), followed by skin injuries (26.1%), with similar patterns observed across both playing positions. It is worth noting that joint/ligament injuries occurred more frequently in backs (42.1%) than in forwards (25.9%). However, the difference between positions was not statistically significant (*p* = 0.283).

Most injuries occurred following contact, both in the overall sample (80.4%) and when analysed by playing position (85.2% in forwards and 73.7% in backs), with no statistically significant difference between positions (*p* = 0.192). Regarding the mechanism of contact, being tackled (33.3%) and open-play collisions (22.7%) accounted for the majority of injuries. Among backs, being tackled was clearly the predominant mechanism, accounting for more than half of all injuries (56.5%). No statistically significant difference was found in the type of contact according to playing position (*p* = 0.094) (Table 2).

Approximately two thirds of all injuries were sustained during the second half (65.2%) (Table 3). Injuries sustained in the second half were more frequent among backs (76.9%) than in forwards (57.5%), but the difference was not statistically significant (*p* = 0.122). As for the pitch location where the injury occurred, the most frequent area was the attacking midfield (33.9% of all injuries), both in forwards and backs. The difference between positions was not statistically significant (*p* = 0.850) (Table 3).

Regarding the body side affected by injuries, the same proportion of injuries was observed on both the right and left sides of the body in the overall sample (48.1%). Forwards sustained more injuries on the left side of the body (48.9%), whereas right-sided injuries were more common among backs (52.9%). This difference was not statistically significant (*p* = 0.371) (Table 3).

A statistically significant difference in injury severity was found according to injury location (*p* = 0.029). Specifically, a statistically significant difference was observed between head and neck injuries and lower-limb injuries (*p* = 0.014), with the latter being associated with a longer recovery period. A statistically significant difference in injury severity was also observed according to injury type (*p* = 0.012). Furthermore, a statistically significant difference was identified between joint/ligament injuries and skin injuries (*p* = 0.001), with the former resulting in a longer recovery period (Table 4).

Regarding the type of contact, scrums were associated with the most severe injuries (M = 23.00 days, SD = 17.09), although this difference was not statistically significant (*p* = 0.299). (Table 4).

There was no statistically significant difference in injury severity according to the timing of the match (*p* = 0.859), with only a slightly higher mean number of days lost observed for injuries sustained during the second half (M = 13.56, SD = 12.68). Regarding body side, a higher mean number of days lost was observed for injuries sustained on the left side of the body (M = 13.89, SD = 13.18). However, this difference was not statistically significant (*p* = 0.371) (Table 5).

## 4. Discussion

The objective of this study was to examine the epidemiology of injuries sustained during the 2023/2024 season of the Portuguese Rugby Union XV top-tier competition. Specifically, the study aimed to explore potential associations between injury characteristics and player positions, as well as between injury features and their respective severity.

The overall injury incidence observed was lower than that reported in the 2022/2023 season for the same competition (54.4 injuries per 1000 player-match-hours, 95% CI: 30.3–96.2) [4]. The injury incidence was also lower than that reported in the Spanish “División de Honor” during the 2018/2019 season, which recorded 53.6 injuries per 1000 player-match-hours (95% CI: 46.7–60.5) [14]. When compared with elite-level rugby, even higher injury incidences are observed. For instance, studies on match injuries during international matches in Scotland and England report values ranging from 78 to 121 injuries per 1000 player-match-hours [5,15,16].

Although 92 injuries were recorded across the season, this number may appear low given the 18 matches played per team. However, it aligns with the total match exposure time and reflects both the inclusion criteria used (e.g., time-loss injuries only) and the natural variability of injury occurrence in a limited sample. Additionally, the possibility of underreporting by medical staff cannot be excluded, which may have led to an underestimation of the true injury burden.

Both injury incidence and injury burden were higher in forwards than in backs. However, the literature is inconsistent, and a definitive association between playing position and overall injury risk cannot be established [17,18,19]. Despite sustaining fewer injuries, backs showed a slightly higher mean injury severity (13.63 days) compared with forwards (12.22 days). The mean severity of all injuries was 12.80 days, which, once again, represents a lower value when compared with elite rugby studies [3,5,15,16].

Several factors may help explain the discrepancies observed between amateur leagues (such as the Portuguese “Top 10”) and elite-level competitions. Leagues such as the English Premiership or the Scottish professional competitions are significantly more competitive and demand higher physical intensity, resulting in greater contact forces during match play [20,21]. Elite medical departments are also more likely to diagnose minor injuries that may be underreported or overlooked in non-professional settings, which may increase the observed injury incidence. Both increased injury frequency and improved diagnostic capacity contribute to higher reported injury incidence. Conversely, more limited access to medical monitoring, potential underestimation of non-acute injuries, and the need for rapid return-to-play decisions due to smaller squad depth may contribute to shorter recorded injury severity in amateur rugby [2,22,23].

More than four out of five injuries occurred following contact (80.4%), and joint/ligament injuries predominated in both forwards and backs, accounting for 32.6% of all injuries, followed by skin injuries, with no statistically significant difference according to playing position. These findings are consistent with those reported in most rugby studies [2,4,6,14,24,25] and may be explained by several factors, including the substantial exposure of both forwards and backs to frequent high-impact collisions, rapid changes in direction, and demanding phases of play such as tackles and rucks. The lower limb was the body segment with the highest proportion of injuries across both playing positions and overall (52.2%), as observed in most of the literature, including a four-year study on match and training injury risk in semi-professional rugby union and a systematic review on the epidemiology of injuries in senior male rugby union sevens [24,25].

Most injuries occurred in the attacking midfield (33.9% of all injuries) and during the second half (65.2%). One possible explanation for the higher proportion of injuries in the second half may relate to the cumulative effects of fatigue, which could impair physical and cognitive performance, potentially affecting reaction time, strength, and technical execution. Similarly, the distribution of injuries across pitch areas may reflect contextual factors such as game intensity, tactical pressure, and frequency of contact situations in specific zones of play. However, these interpretations should be considered as hypotheses, as fatigue levels and contextual match demands were not directly measured in this study [26,27].

Although certain trends were observed in the distribution of injuries—such as the predominance of lower-limb injuries, a higher frequency during the second half, or increased injury occurrence in the attacking midfield—it is important to note that many of these variables (e.g., anatomical location, type of injury, injury mechanism, type of contact, match time, pitch location, and body side) did not reach statistical significance in this study. Therefore, while these patterns are consistent with previous literature and may provide useful insights for injury prevention, they should be interpreted with caution and not considered definitive findings. These observations may warrant further investigation in studies with larger sample sizes and greater statistical power.

The lower limb was associated with a longer recovery period (15.56 days on average). This could be attributed to the fact that, in this study, the lower limb was not subdivided, meaning that all injuries occurring in the thighs, groin, knee, ankle, and foot regions were collectively categorized as “lower-limb” injuries. Given that the lower limb is likely the most involved body region during a rugby match, being heavily involved in running, kicking, sidestepping, or absorbing impact during tackles, it is naturally more susceptible to injury. The high severity observed in lower-limb injuries can be explained by the prolonged recovery periods required for some of the most common injuries in this region, such as ligament ruptures, meniscal tears, and fractures. [28,29] In this study, bone fractures accounted for the highest average injury severity (21.83 days), which may have contributed to the higher injury severity observed in the lower limb.

Scrums were associated with the highest injury severity (23 days on average) compared to other events, although this difference was not statistically significant. This may be due to the small sample size, as only three scrum-related injuries were reported, and a single case with exceptionally high severity could have disproportionately influenced the results. While injuries were more frequent in the second half, as previously mentioned, the severity of injuries sustained in both halves was relatively similar (approximately 13 days on average). Although specific data comparing injury severity between halves is limited, a study conducted in Spain contradicted our findings, showing that the most severe injuries occurred in the first half [18]. Therefore, it remains challenging to draw a strong association between injury severity and the timing of injury occurrence during matches.

No significant difference was observed in injury severity according to the injured body side, and the available data remain insufficient to establish definitive associations between these variables. Injuries sustained in the attacking 22 m were associated with a longer recovery period. This result aligns with the premise that, closer to the try line, match situations tend to involve more physically intense confrontations. The urgency to score a try also leads to higher intensity, riskier plays, and fatigue, making players more susceptible to injury [26,30]. While the relationship between accumulated exposure and injury risk remains an important topic in rugby and other high-intensity sports, further research with more robust study designs is needed to clarify the potential impact of workload on injury outcomes [31,32].

Although several comparisons did not reach statistical significance, some observed differences between groups showed relevant percentage variations that should not be disregarded. This may partly reflect limited statistical power due to the relatively small sample size and the reduced number of participating teams, particularly in subgroup analyses according to playing position and injury characteristics. Descriptive differences in injury patterns were observed between forwards and backs. However, these differences did not reach statistical significance. As a result, the possibility of a Type II error cannot be excluded, and some non-significant findings may still be clinically relevant. Therefore, these results should be interpreted with caution, considering both statistical significance and epidemiological relevance within the context of match injury surveillance in rugby.

### Limitations

This study has several limitations that should be considered when interpreting the findings.

First, only five of the ten teams competing in the Portuguese Senior Male Rugby Union XV Championship participated in the surveillance programme, and data collection was further reduced during the playoff phase. Consequently, the sample may not fully represent the national competition, and the generalisability of the findings should be interpreted with caution.

Second, injury diagnoses, classifications, and return-to-play decisions were performed by the medical staff of each participating club rather than by a centralised assessment team. Although all healthcare professionals received standardised preseason instructions regarding injury definitions, classification criteria, and reporting procedures, formal inter-rater reliability was not assessed. In addition, the composition and expertise of medical teams differed between clubs, which may have introduced variability in injury diagnosis, injury classification, and return-to-play decisions. The observed frequency of concussion highlights the importance of maintaining robust concussion recognition, assessment, and management strategies in accordance with current international Rugby Union recommendations. However, adherence to concussion protocols was not evaluated in the present study.

Third, detailed diagnostic procedures and imaging confirmation were not collected. Therefore, it cannot be determined whether some observed differences reflected true epidemiological variation or differences in clinical assessment and management between teams.

Fourth, match exposure was estimated assuming full participation of all fifteen players throughout each match because reliable individual time-on-field data were unavailable. Although this approach is consistent with previous epidemiological studies and was applied uniformly across all the participating teams, it may have led to slight overestimation of exposure and, consequently, underestimation of injury incidence.

Finally, only match-related injuries were included in the surveillance system. Training exposure and training-related injuries were not recorded, preventing assessment of the overall injury burden and limiting interpretation of the relationship between workload and injury occurrence. Future epidemiological studies should incorporate comprehensive surveillance systems including both match and training exposure, standardised diagnostic procedures, centralised case verification, and participation from all clubs competing at the national level.

## 5. Conclusions

This study provides an updated prospective epidemiological analysis of injuries sustained during the 2023/2024 season in Portugal’s highest national amateur Rugby Union competition, complementing previous national surveillance data. Match injuries predominantly affected the lower limbs and were mainly sustained through contact mechanisms, particularly during tackles, with a greater proportion occurring in the second half of matches. Injury patterns also differed according to playing position, reflecting the distinct physical and tactical demands of forwards and backs.

These findings contribute to the current understanding of match-related injury epidemiology in top-tier Portuguese rugby and provide context-specific information that may support the development of targeted injury prevention strategies. However, the findings should be interpreted within the scope of match exposure only, as training exposure and training-related injuries were not included in the surveillance system.

Future research should incorporate comprehensive injury surveillance across both training and match environments, include multiple competitive seasons and a larger number of teams, and further investigate the factors associated with injury occurrence to strengthen the evidence base for injury prevention in Portuguese Rugby Union.

## Figures and Tables

**Table 1:** Injury incidence, mean severity and burden.

	Total Injuries n (%)	Incidence (95% CI)	Mean Severity d (SD)	Burden (95% CI)
Total	92	49.46 (39.78–59.68)	12.80 (12.17)	633.33 (597.31–670.97)
Forwards	54 (58.7)	54.44 (40.32–69.56)	12.22 (12.54)	665.32 (614.92–714.72)
Backs	38 (41.3)	43.78 (29.95–57.60)	13.63 (11.74)	596.77 (546.08–647.47)

Note: n = absolute frequency; d = days; SD = Standard Deviation.

**Table 2:** Anatomical location, type of injury and relation with contact according to player position.

	All Players (n = 92)	Forwards (n = 54)	Backs (n = 38)	*p*
Anatomical location n (%)				0.329
Head and neck	14 (15.2)	10 (18.5)	4 (10.5)	
Upper limb	6 (6.5)	2 (3.7)	4 (10.5)	
Trunk	24 (26.1)	16 (29.6)	8 (21.1)	
Lower limb	48 (52.2)	26 (48.1)	22 (57.9)	
Type of injury n (%)				0.283
Joint/ligament	30 (32.6)	14 (25.9)	16 (42.1)	
Muscle/tendon	21 (22.8)	13 (24.1)	8 (21.1)	
Skin	24 (26.1)	13 (24.1)	11 (28.9)	
Bone	6 (6.5)	5 (9.3)	1 (2.6)	
Concussion	3 (3.3)	2 (3.7)	1 (2.6)	
Other	8 (8.7)	7 (13.0)	1 (2.6)	
Injury after contact n (%)				0.192
No	18 (19.6)	8 (14.8)	10 (26.3)	
Yes	74 (80.4)	46 (85.2)	28 (73.7)	
Type of contact n (%)				0.094
Collision	15 (22.7)	9 (20.9)	6 (26.1)	
Being tackled	22 (33.3)	9 (20.9)	13 (56.5)	
Tackling	13 (19.7)	11 (25.6)	2 (8.7)	
Ruck	6 (9.1)	5 (11.6)	1 (4.3)	
Line-Out	1 (1.5)	1 (2.3)	0 (0.0)	
Maul	2 (3.0)	2 (4.7)	0 (0.0)	
Scrum	3 (4.5)	3 (7.0)	0 (0.0)	
Other	4 (6.1)	3 (7.0)	1 (4.3)	

Note: n = absolute frequency.

**Table 3:** Time of match, pitch location and body side according to player position.

	All Players (n = 92)	Forwards (n = 54)	Backs (n = 38)	*p*
Time of match n (%)				0.122
First half	23 (34.8)	17 (42.5)	6 (23.1)	
Second half	43 (65.2)	23 (57.5)	20 (76.9)	
Pitch location n (%)				0.850
Defensive 22	7 (12.5)	5 (15.6)	2 (8.3)	
Defensive midfield	16 (28.6)	9 (28.1)	7 (29.2)	
Attacking midfield	19 (33.9)	11 (34.4)	8 (33.3)	
Attacking 22	14 (25.0)	7 (21.9)	7 (29.2)	
Body side n (%)				0.371
Right	38 (48.1)	20 (44.4)	18 (52.9)	
Left	38 (48.1)	22 (48.9)	16 (47.1)	
Both	3 (3.8)	3 (6.7)	0 (0.0)	

Note: n = absolute frequency.

**Table 4:** Injury severity according to anatomical location, type of injury, type of contact.

	Mean Severity	
	M (SD) (n = 92)	*p*
Anatomical location		0.029
Head and neck	6.71 (6.99)	
Upper limb	8.67 (5.54)	
Trunk	11.88 (8.91)	
Lower limb	15.56 (14.48)	
Type of injury		0.012
Joint/ligament	16.67 (12.18)	
Muscle/tendon	12.62 (10.08)	
Skin	6.50 (3.60)	
Bone	21.83 (22.03)	
Concussion	12.67 (7.23)	
Other	11.00 (18.51)	
Type of contact		0.299
Collision	10.60 (12.01)	
Being tackled	10.73 (9.84)	
Tackling	7.85 (2.58)	
Ruck	17.33 (10.11)	
Line-Out	14.00 (---)	
Maul	6.00 (8.49)	
Scrum	23.00 (17.09)	
Other	11.00 (12.68)	

Note: M = Mean; SD = Standard Deviation.

**Table 5:** Injury burden according to time of match, pitch location and body side.

	Injury Burden	
	M (SD) (n = 92)	*p*
Time of match		0.859
First half	13.00 (10.89)	
Second half	13.56 (12.68)	
Body side n (%)		0.371
Right	13.29 (10.25)	
Left	13.89 (13.18)	
Both	4.00 (1.00)	

Note: M = Mean; SD = Standard Deviation.

## Data Availability

The data presented in this study are available upon request from the corresponding author due to privacy and ethical restrictions.

## Abbreviations

The following abbreviations are used in this manuscript:

M	Mean
SD	Standard Deviation
CI	Confidence Interval
